# Predictors of metabolic monitoring among schizophrenia patients with a new episode of second-generation antipsychotic use in the Veterans Health Administration

**DOI:** 10.1186/1471-244X-9-80

**Published:** 2009-12-18

**Authors:** Lizheng Shi, Haya Ascher-Svanum, Yi-Ju Chiang, Yingnan Zhao, Vivian Fonseca, Daniel Winstead

**Affiliations:** 1 School of Public Health and Tropical Medicine, Tulane University, 1440 Canal Street, New Orleans, LA 70112, USA; 2 School of Medicine, Tulane University, 1430 Tulane Avenue, New Orleans, LA 70112, USA; 3 Southeast Louisiana Veterans Health Care System, 1601 Perdido Street, New Orleans, LA 70112, USA; 4 US Outcomes Research, Eli Lilly and Company, Lilly Corporate Center, DC 4133, Indianapolis, Indiana 46285, USA

## Abstract

**Background:**

To examine the baseline metabolic monitoring (MetMon) for second generation antipsychotics (SGA) among patients with schizophrenia in the Veterans Integrated Service Network (VISN) 16 of the Veterans Health Administration (VHA).

**Methods:**

VISN16 electronic medical records for 10/2002-08/2005 were used to identify patients with schizophrenia who received a new episode of SGA treatment after 10/2003, in which the VISN 16 baseline MetMon program was implemented. Patients who underwent MetMon (MetMon+: either blood glucose or lipid testing records) were compared with patients who did not (MetMon-), on patient characteristics and resource utilization in the year prior to index treatment episode. A parsimonious logistic regression was used to identify predictors for MetMon+ with adjusted odds ratios (OR) and 95% confidence intervals (CI).

**Results:**

Out of 4,709 patients, 3,568 (75.8%) underwent the baseline MetMon. Compared with the MetMon- group, the MetMon+ patients were found more likely to have baseline diagnoses or mediations for diabetes (OR [CI]: 2.336 [1.846-2.955]), dyslipidemia (2.439 [2.029-2.932]), and hypertension (1.497 [1.287-1.743]), substance use disorders (1.460 [1.257-1.696]), or to be recorded as obesity (2.052 [1.724-2.443]). Increased likelihood for monitoring were positively associated with number of antipsychotics during the previous year (FGA: 1.434 [1.129-1.821]; SGA: 1.503 [1.290-1.751]). Other significant predictors for monitoring were more augmentation episodes (1.580 [1.145-2.179]), more outpatient visits (1.007 [1.002-1.013])), hospitalization days (1.011 [1.007-1.015]), and longer duration of antipsychotic use (1.001 [1.001-1.001]). Among the MetMon+ group, approximately 38.9% patient had metabolic syndrome.

**Discussion:**

This wide time window of 180 days, although congruent with the VHA guidelines for the baseline MetMon process, needs to be re-evaluated and narrowed down, so that optimally the monitoring event occurs at the time of receiving a new episode of SGA treatment. Future research will examine whether or not patients prescribed an SGA are assessed for metabolic syndrome following the index episode of antipsychotic therapy, and whether or not such baseline and follow-up monitoring programs in routine care are cost-effective.

**Conclusion:**

The baseline MetMon has been performed for a majority of the VISN 16 patients with schizophrenia prior to index SGA over the study period. Compared with MetMon- group, MetMon+ patients were more likely to be obese and manifest a more severe illness profile.

## Background

It has long been known that psychiatric patients experience increased morbidity and mortality associated with a range of physical disorders including diabetes and cardiovascular disorders (CVD) [1]. Individuals with schizophrenia are more likely to be overweight or obese than the population at large [2-4]. Obesity further increases patients' risk of cardiovascular morbidity and mortality. Before the introduction of the antipsychotic drugs, it was acknowledged that patients with schizophrenia may be at a higher risk to develop diabetes (overall prevalence of diabetes of 12.9% to 18.9%) than the general population in similar age groups without schizophrenia (a prevalence of 3% to 5.5%) [5,6]. Among people with schizophrenia, there is a high prevalence of obesity, which is known as a risk factor for diabetes [7]. Further, the results of a recent study showed that compared with the general population, people with major mental illness lose an average of more than 25 years of potential life, primarily due to cardiovascular disease [8].

Second-generation antipsychotics (SGA) or atypical drugs have gained widespread acceptance for the management of patients with schizophrenia and have been demonstrated to have some advantages over first-generation antipsychotics (FGA) or typical drugs. However, treatment with several SGAs has been associated with increased prevalence of diabetes, obesity, and dyslipidemia [9], which are risk factors for cardiovascular diseases [10-12].

Several expert consensus guidelines calling for routine monitoring of weight, lipids, and glucose have been developed in the recent years such as those from the Mount Sinai Conference on Medical Monitoring [13] and the American Diabetes Association/American Psychiatric Association Consensus Development Conference on Antipsychotic Drugs and Obesity and Diabetes [14]. Despite the availability of the census guidelines, mentally ill patients often do not receive adequate recognition of, monitoring of, or care for their physical health conditions. It is clear that patients across settings are not being adequately monitored for the presence of metabolic disease [15]. A retrospective cohort study of 4 state Medicaid programs (California, Oregon, Tennessee, and Utah) indicated that glucose and lipid screenings were underutilized in patients receiving a new episode of SGA drug therapy (< 20% baseline glucose testing and < 10% lipid testing) [16]. A Canadian chart review study using the HIV clinic as a control for monitoring of hypertension, diabetes, and dyslipidemia reported that metabolic risk factors were less frequently documented in mental health clinics [17].

Current concern about the need to enhance the quality of health care for mental illness and the availability of consensus guidelines have led clinicians, researchers, and administrators in the VISN 16 network (the largest network among all 22 VHA VISNs) to develop the first VHA routine baseline metabolic monitoring (MetMon) program for metabolic safety in patients treated with antipsychotic drugs as a network-wide performance measure in October 2003 [[18], detailed data available upon request]. The VISN 16 mental health leaders worked together to define measures for performance of guideline-recommended baseline monitoring at the time of new antipsychotic starts. Specifically, in Fiscal Year (FY04), the VISN 16 Mental Health Product Line implemented 2 performance measures: 1) the proportion of patients at each facility that were newly started on an antipsychotic and had weight recorded within 180 days prior to or 30 days after the new antipsychotic was prescribed; and 2) the proportion of patients that had blood glucose monitored in the same time frame. A performance measure for baseline lipid monitoring was added in FY05. The fully successful level for a VA facility was defined as 85% in FY04 and 90% in FY05 for weight, 75% to 78% for glucose, and 70% in FY05 for lipids respectively. With a sample size of 4,066 patients with new antipsychotic starts in FY03, and 4,019 in FY04, the average facility rate of weight monitoring was 87.9% in 2003 and 92.2% in FY04, with 8 out of 10 facilities performing at the "fully successful" level. In FY04, 8 facilities were "fully successful" at implementing the blood glucose performance measure. The rate of baseline lipid monitoring in FY05 was 63.3% (compared with 59.3% in FY04). Overall, implementation of the baseline MetMon has reduced the variations in the VISN 16 health system factors such as settings and lack of access, which could be attributed to the reasons why some patients are not monitored, and provided a unique opportunity to focus on the patient-level characteristics in our study.

In an effort to improve patient care by increasing metabolic monitoring, this study sought to identify patient characteristics that are associated with decreased baseline MetMon. Specifically, this retrospective study among VISN 16 patients with schizophrenia who received a new episode of SGA treatment aimed to identify predictors (patient demographic and illness characteristics, antipsychotic use patterns, and health care resource utilization) of undergoing the baseline MetMon.

## Methods

After obtaining Tulane/VHA IRB and VHA Research and Development (R&D) approvals for this study protocol, the VISN 16 data warehouse was used to evaluate antipsychotic medication patterns and health outcomes. We examined metabolic monitoring in a retrospective cohort study in patients with schizophrenia receiving care from the Veterans Health Administration, US Department of Veterans Affairs (VA) which is an integrated health system providing comprehensive health care services. Patients' demographic information, inpatient care, outpatient care, outpatient pharmacy records, vital signs, and laboratory results during the period of October 2002 through September 2005 in VISN 16 were extracted from the VISN 16 data warehouse. Data format and content which was stripped of patient identifiers was in compliance with the Health Insurance Portability and Accountability Act (HIPAA) requirements.

The baseline metabolic monitoring services in the study were required to occur within 180 days prior to a new SGA treatment episode (i.e., the index date). The evaluation method had intentionally excluded the first 30 days after the new episode of SGA was prescribed to avoid potential misattribution of pre-existing metabolic syndrome to the index drug. It was previously shown that rapid weight gain can occur early on - within the first 2-3 weeks of treatment - particularly with certain SGA such as olanzapine [19,20]. Such rapid and early weight gain could lead to increase the probability that some patients who did not have a pre-existing metabolic syndrome might have met the metabolic syndrome criteria within the first 30 days after a new treatment episode. Due to a retrospective study design, it was hard to ascertain whether lab testing belonged to baseline monitoring or follow-up care during the first month following the prescribed index SGA was filled by patients.

Treatment episode with any SGA reflected 3 different types of treatment episodes: switch, new start, and augmentation. The switch episode was defined by the discontinuation of the previous antipsychotic agent within 60 days after the index date. A new start episode was defined by receiving a new episode of antipsychotic agent after a medication break of at least 60 days. An augmentation episode was defined by the concurrent use of the new antipsychotic agent and the previous antipsychotics for longer than 60 days. Only was the first antipsychotic episode included if there were multiple episodes for a patient.

Additional file 1 presents the process leading to the analytical sample. We initially identified 8,816 patients who had at least 1 record of antipsychotic prescription after October 2003 in order to ensure that all patients at least had 1 year before the earliest possible index date (October 2002-September 2003). All patients also had a diagnosis of schizophrenia based on the codes of the International Classification of Diseases, 9th Revision, Clinical Modification (ICD-9-CM: 295.xx) for either at least 1 inpatient service or at least 2 outpatient visits. Patients were excluded if they were diagnosed with a bipolar disorder (ICD-9-CM code: 296.0x, 296.4x, 296.5x, 296.6x, 296.7x, 296.8x, 296.9x) or any type of dementia (ICD-9-CM code: 294.1x, 290.4x). We excluded non-treatment episodes (n = 2,354) who had been on only 1 antipsychotic drug without any break in therapy for more than 30 days during the whole study period. These excluded patients may not present as the target candidates for the VISN 16 antipsychotic monitoring program because the baseline MetMon required a new treatment episode. Therefore, a total of 6,462 patients had at least 1 new treatment episode, starting from the index date when the patient started a new antipsychotic agent. We further excluded patients who only had 1 filled prescription (n = 393), received more than 2 index antipsychotic drugs (n = 677), or switched to an FGA agent (n = 683).

The analytical sample of 4,709 patients was used to identify 2 comparison cohorts. One cohort (n = 3,568) included patients who received the baseline MetMon (at least 1 claims record or medical record of blood glucose testing or lipid panel) within 6 months before the index date (MetMon+) and another cohort (n = 1,141) included those who did not (MetMon-). All treatment episodes were further classified by type of episodes (switch, new start, and augmentation).

The MetMon+ and MetMon- groups were compared on several variables assessed during the pre-existing year (i.e., during the year prior to starting a new antipsychotic episode). These variables included the socio-demographics (age, gender, race, and insurance coverage other than VHA), illness characteristics including the Charlson Comorbidity Index (CCI) based on ICD-9-CM codes [21] and ICD-9-CM diagnosis of substance (drug and/or alcohol) dependence disorders, antipsychotic medication use patterns in the pre-existing year including the duration and medication possession ratio (MPR) of antipsychotic agents, numbers of SGAs, and use of SGA+FGA combination. We also compared the 2 cohorts on outpatient visits (psychiatric or nonpsychiatric) during 1 year prior to the index date. For inpatient care records, the count of hospital admission as a confounder was examined during the 180 days before receiving the baseline MetMon services because glucose and lipid testing may be more likely to have been performed during a recent hospitalization over the same timeframe of 6 months than otherwise.

For patients who underwent the baseline MetMon, we also examined 5 cardio-metabolic risk factors: (1) presence of serum high density lipoprotein (HDL) cholesterol level <40 mg/dl (male) or <50 mg/dl (female); (2) fasting plasma glucose (FPG) level of at least 110 mg/dl; (3) serum triglyceride (TC) level of at least 150 mg/dl; (4) blood pressure of at least 130/85 mm/Hg; and (5) waist circumference of more than 102 cm (>40 inches) for men and >88 cm (>35 inches) for women. Since waist circumference was rarely reported in the medical records, we replaced it with a body mass index (BMI) ≥ 28.8 because it was found to be correlated with a waist circumference of 102 cm for men and 88 cm for women [22,23]. In addition, we further reported a recent BMI in 4 categories: underweight (<18.5), normal (18.5-24.9), overweight (25.0-29.9), and obese (≥ 30.0), which is the weight categorization used by the National Heart, Lung, and Blood Institute [24]. For those without the baseline MetMon, we reported their most recent values of blood pressure and BMI prior to the index date. Using the lab testing-based indicators, we created a dichotomous variable reflecting whether a patient met the criteria for metabolic syndrome as defined by the National Cholesterol Education Program (NCEP) (at least 3 out of 5 parameters) or not (2 or less indicators) in 2 methods. To meet the metabolic syndrome using method 1, it was required to include patients who had all 5 indicators with no missing lab tests. Furthermore, while keeping the same sample as method 1, method 2 was used to expand the lab testing-based indicators to the composite indicators, which include all 3 types of records: lab results, diagnosis codes (diabetes, dyslipidemia, hypertension), and medication uses (antidiabetics, lipid-lowering drugs, and antihypertensives). Because the NCEP criteria has 2 lipid-related indicators (HDL and triglyceride), to be conservative, either dyslipidemia diagnosis or lipid-lowering drugs for a patient was counted as only 1 positive lipid indicator rather than 2.

The variables, in which the 2 cohorts were shown to significantly differ in the year prior to the index date through t-tests or chi-square tests, were then fit into a parsimonious logistic regression to identify predictors for undergoing the baseline MetMon. Specifically, a step-wise progression method was used to select the most parsimonious multivariate model. All analyses were performed using the SAS version 9.13. The statistical significance was set at the level of 0.05.

## Results

Among 4,709 patients in the analytical sample, 75.77% (n = 3,568) received the baseline MetMon during the 180 days before the new antipsychotic episode. The MetMon+ group had 2,815 patients (78.90%) with new start episodes, 439 patients (12.30%) with switch episodes, and 314 patients (8.80%) with augmentation episodes. The MetMon- group had 986 patients (86.42%) with new start episodes, 97 (8.50%) with switch episodes, and 58 with augmentation episodes (5.08%).

Additional file 2 presents the baseline demographic and illness characteristics for these 2 cohorts. Although the MetMon- and MetMon+ groups did not significantly differ in gender or ethnicity, the MetMon+ patients were more likely to have non-VHA insurance coverage, higher CCI scores, diagnoses of diabetes, dyslipidemia, hypertension, or substance use disorders, more outpatient office visits (psychiatric, nonpsychiatric, and either) during the prior year, more hospital admissions during the 180 days before the start of a treatment episode, and were significantly older. The hospitalization days for the MetMon+ group was twice as long as that for the MetMon- group (12.96 days vs. 6.37 days, p < .0001). Finally, there was a significant difference in the MetMon rates across 3 calendar years for each index date.

Additional file 3 summarizes the pre-existing antipsychotic use patterns for the 2 cohorts. The MetMon+ group had been intensively treated by antipsychotic agents. These patients were associated with significantly longer treatment duration, a higher medication possession ratio, and a higher number of different antipsychotic drugs.

Additional file 4 presents the cardio-metabolic risk factors for the 2 cohorts. The MetMon+ group had a significantly higher rate of BMI ≥ 28.8 (39.43% vs. 18.58%, p < .0001), while the rate of high blood pressure was comparable across comparison cohorts. MetMon+ patients had met at least 3 criteria out of 5 parameters for metabolic syndrome in the pre-existing period. The prevalence rates of metabolic syndrome among the MetMon+ group varied with different methods: method 1 (22.69%) and method 2 (38.89%). Using the method 2, 7.19% MetMon- patients can be also classified to have metabolic syndrome, based on the diagnosis and medication use information.

Additional file 5 presents a parsimonious logistic regression model (in adjusted odds ratio [95% confidence interval]) to identify the predictors of undergoing the baseline metabolic monitoring services. Having the baseline diagnosis or medication use for diabetes, dyslipidemia, hypertension, or a higher BMI (i.e., BMI ≥ 28.8) was among the strongest predictors for undergoing the baseline MetMon. Specially, baseline dyslipidemia, diabetes and higher BMI almost more than doubled the likelihood of undergoing the baseline MetMon (dyslipidemia: 2.439 [2.029-2.932]), diabetes 2.336 [1.846-2.955]), high BMI (2.052 [1.724-2.443]). A comorbidity condition of baseline hypertension increased the likelihood of baseline monitoring by approximately 50% (1.497 [1.287-1.743]). Patients with a diagnosis of substance dependence were 46.0% more likely to undergo the baseline MetMon (1.460 [1.257-1.696]). Augmentation episode - a marker of increased treatment complexity - was associated with a 58% higher likelihood of undergoing the baseline MetMon (1.580 [1.145-2.179]). For each additional SGA used, patients were 50% more likely to be monitored (1.503 [1.290-1.751]), while for each additional FGA, an increase in receiving MetMon was 43% higher (1.434 [1.129-1.821]). Other significant predictors included the number of non-psychiatric outpatient office visits, hospitalization days, and the treatment duration with the most recent antipsychotic agent, but they increased the likelihood of monitoring by no more than 10%.

## Discussion

The baseline metabolic monitoring program at the VHA's largest region - VISN 16 - is an experiment with important public health implications for disease management of patients with schizophrenia. This monitoring program was associated with a high rate (76%) of the baseline MetMon for patients with schizophrenia who received a new treatment episode of SGA. The MetMon rate and sample size presented by this study are very similar to, and yet slightly lower than, the VISN internal report, reflecting that the first month post-index was not used to calculate the baseline MetMon. It is also well known that the VHA population is relatively older than a Medicaid or a commercially insured population. Metabolic screening is more likely to occur in older adults therefore one would expect average VHA rates of screening to be higher than average rates of screening in Medicaid or commercially insured patients. Furthermore, a much higher baseline MetMon rate reported in this study is not surprising in the environment where the MetMon was required by the VISN 16 Mental Health Product Line.

Despite a high rate of monitoring, patients who were not monitored were significantly different from monitored patients on a host of demographic, clinical, and resource use variables. The monitored patients were as more than twice as likely to be overweight or obese. The monitored patients were also associated with baseline diagnoses and treatments for diabetes, dyslipidemia, or hypertension, a higher rate of substance use disorders, more outpatient visits, a longer duration of antipsychotic use, and had previously used a larger number of different antipsychotic drugs. Current findings suggest that the baseline MetMon tends to take place among patients manifesting a more severe illness profile and for those who may be considered by their clinicians to be at a greater risk of metabolic syndrome due to being overweight or obese or having pre-existing conditions. Interestingly, the monitored group was not significantly more likely to be treated with any specific SGA prior to new treatment episode of another SGA.

The logistical regression model suggests that among the 11 variables that significantly differed between patients with versus without the baseline MetMon, there was a small and distinct set of 8 variables that best predicted undergoing the monitoring process prior to on the index date of SGA. These are 3 pre-existing metabolic conditions, higher BMI, augmentation with another SGA, multiple antipsychotic drugs, diagnosis of substance dependence, and psychiatric hospitalization prior to the index date of SGA. Importantly, the presence of pre-existing conditions or obesity (BMI ≥ 28.8) was among the set of strongest predictors, as each of them almost doubled the likelihood of undergoing the baseline MetMon. However, the disparities between the groups were not significantly related to either race or gender.

Current findings also show that among the monitored patients, approximately 38.9% of MetMon+ patients were identified as having metabolic syndrome as defined by the NCEP using the composite indicators. This rate is very close to the one previously found for patients with schizophrenia. Results from a large randomized, double-blind study sponsored by the National Institute of Mental Health (NIMH) - the Clinical Antipsychotic Trials of Intervention Effectiveness (CATIE) found that at baseline 40.9% (of 689 participants) had a metabolic syndrome based on the NCEP criteria and 42.7% based on the FPG ≥ 100 mg/dl [15]. In addition to the difference in monitoring rate (per-protocol 100% in the CATIE study and 76% of this study), the other reasons for the observed difference are unclear, but may be related to differences in methodology, because the VHA patients, despite undergoing the baseline MetMon in the 180 days prior to the index date, could have developed a metabolic syndrome during the relatively long ensuing time between the monitoring event and the new treatment episode. This wide time window of 180 days, although congruent with VHA guidelines for the routine baseline MetMon process, needs to be re-evaluated and narrowed down, so that optimally the monitoring event occurs at the index date. Although the NCEP definition of "metabolic syndrome" includes an FPG of ≥ 110 mg/dl, the ADA has since changed their threshold for a diagnosis of impaired fasting glucose to at least 100 mg/dl [25,26]. This new cutpoint should be applicable to this study for identifying the lower FPG boundary as 1 criterion for metabolic syndrome. The MetMon was defined as glucose or lipid testing. Based on Additional file 4, all MetMon+ subjects had glucose testing and 80% of those also had lipid testing. The pattern may reflect the sequential approach of implementing the monitoring (weight and glucose in FY04 and all monitoring in FY05). It would be interesting to know the characteristics of those patients who did not get the lipid testing in the future research. Furthermore, caution is needed in the interpretation of the reported rate of metabolic syndrome among the MetSyn- patents, which is very low because of no lab testing for this group.

Further studies on the management of schizophrenia with comorbid metabolic syndrome are also warranted so that effective interventions may be developed to improve the quality of care for this particular group of patients at high risk of cardiovascular diseases. By routinely performing physical health monitoring, referrals, and/or treatment for patients with schizophrenia, the current system of fragmented mental and physical health services could be transformed into a system focusing on early interventions [27].

To our knowledge, there are no published studies on the rate of baseline MetMon for patients with schizophrenia in a large health care system after the guideline-based baseline MetMon has been implemented as a performance indicator, nor is the information currently available on the proportion of patients who are identified via the baseline MetMon to have a metabolic syndrome. No disparities in the monitoring services have existed in terms of gender and race. Age disparity in the univariate analysis appeared to be due to the large sample size for each comparison cohort. The baseline MetMon was designed as a VISN 16 network quality improvement indicator, yet there were still about 25% patients who did not receive the baseline MetMon. In clinical practice, the MetMon has been found very low in both Medicaid and privately insured populations [16,28,29]. The inclusion of BMI as an indicator of who received testing and who did not is quite unique for this study. This measure has not been available for study in the previous studies examining Medicaid and commercially-insured patients, and the VA results indicate that BMI is a strong risk determinant of who received testing. Beyond the issue of whether or not the patients have received the appropriate tests before a new treatment episode of SGA, a more important issue for future research is whether or not patients prescribed an SGA are assessed for metabolic syndrome following the new episode of drug therapy, and whether or not such baseline and follow-up monitoring programs in the routine care are cost-effective. In addition to those patient-level factors found in this study, the factors (either barriers or facilitators) at the facility or system level would require further clarification in future research.

Current findings need to be evaluated in the context of the study's limitations. First, the definition of "baseline" per VHA guidelines could be up to 6 months prior to the index date. As noted earlier, this time frame is too long, likely allowing for the development of a metabolic syndrome during the ensuing time between the "baseline" MetMon and the index date. Second is our reliance on retrospective claims database analyses to assess the baseline MetMon and presence of a metabolic syndrome. Although the VA received care from the VA system as an integrated system, patients with non-VA insurance whose data on lab testing ordered by a non-VA provider were not necessarily linked with the study dataset unless the records were submitted into the VA as patient's medical records. We are not clear about the magnitude of underreporting as a source of misclassification bias. A prospective study would have been preferable as it would have yielded more complete and accurate data. Considering the high cost of conducting large prospective studies, another potential approach for future research may involve the use of the VHA electronic medical records (EMR) as a data collection tool for a prospective observational study. These records have more detailed and relevant clinical information, which is rarely captured in claims-based databases. A third limitation is our use of BMI instead of waist circumference, the metabolic parameter required in the NCEP definition of a metabolic syndrome [26]. Fourth, the indicators for metabolic syndrome were not collected during the same clinic visit or at least recorded at the same date in the database. We allowed all metabolic parameters to be tested within 6 months before the start of a new SGA episode and selected the results of the ones closest to the index date. Finally, our analysis focused on patient-level factors whereas other factors (either barriers or facilitators) at the facility or system level could have also impacted the likelihood of undergoing the baseline MetMon, despite the quality improvement effort of the monitoring program.

## Conclusion

The current analysis of the baseline MetMon program at the VHA's largest region (VINS 16) has found that a majority of patients with schizophrenia who received a new episode of SGA treatment had undergone the baseline MetMon services prior to the index treatment. However, 25% of the patients were not monitored. The monitored patients were, however, significantly different from those who were not monitored on a host of baseline demographic and clinical characteristics as well as on prior use of health care services. Among these variables, the set of best predictors of undergoing the baseline MetMon were pre-existing related conditions and obesity. Findings highlight the need for routine monitoring of weight, lipids, and glucose in a systematic manner for all persons receiving a new episode of antipsychotic treatment, regardless of body weight, illness profile, or use of health care services, and to do so at the outset of new episode.

## Competing interests

Dr. Shi, Ms. Chiang, and Ms. Zhao are affiliated with the Department of Health Systems Management, School of Public Health and Tropical Medicine, Tulane University, 1440 Canal Street, Suite 1900, New Orleans, LA 70112 (e-mail: lshi1@tulane.edu). Dr. Shi and Ms. Zhao are also a WOC employee/investigator for the Southeast Louisiana Veterans Health Care System. At the project initiation, Dr. Shi was a WOC employee/investigator for the Little Rock Veterans Health Care System. Dr. Fonseca and Dr. Winstead are with School of Medicine Tulane University. Dr. Ascher-Svanum is with Eli Lilly and Company, Indianapolis, Indiana.

## Authors' contributions

LS was the principal investigator for the study (conceptualization, study design, analytical plan, SAS programming guidance, interpretation of the results, and manuscript preparation). HA-S conceived of the study, participated in its design, the analytical plan, the interpretation of the results, and helped draft the manuscript. Y-JC performed the statistical analyses, and participated in the design of the study, the analytical plan, and the interpretation of the results. YZ performed the additional statistical analyses and the interpretation of the results. VF and DW participated in the design of the study, the analytical plan, the interpretation of the results, and assisted in drafting the manuscript. All authors read and approved the final manuscript.

## Pre-publication history

The pre-publication history for this paper can be accessed here:

http://www.biomedcentral.com/1471-244X/9/80/prepub

## Supplementary Material

Additional file 1 **Table 1: Patient selection based on the antipsychotic prescription refilling records**. The patient selection process for data analysis. Click here for file

Additional file 2 **Table 2: Baseline characteristics and health services utilization for schizophrenia patients with versus without baseline monitoring**. Cross-cohort comparisons of baseline characteristics and health services utilization. Click here for file

Additional file 3 **Table 3: Baseline antipsychotic use patterns for schizophrenia patients with versus without baseline antipsychotic metabolic monitoring**. Cross-cohort comparisons of use patterns of baseline antipsychotic agents. Click here for file

Additional file 4 **Table 4: Baseline metabolic syndrome parameters among patients with schizophrenia receiving a new treatment episode of SGA**. Cross-cohort comparisons of baseline metabolic syndrome parameters. Click here for file

Additional file 5 **Table 5: Logistic regression stepwise model to identify predictors of undergoing baseline antipsychotic metabolic monitoring**. Influencing factors identified using a logistic regression on whether a patient receiving baseline antipsychotic metabolic monitoring. Click here for file
