# Supplementary material for: Predictors of metabolic monitoring among schizophrenia patients with a new episode of second-generation antipsychotic use in the Veterans Health Administration
Source: BMC Psychiatry. 2009 Dec 18;9:80. doi: 10.1186/1471-244X-9-80 (PMC2807859; doi:10.1186/1471-244X-9-80)
Supplement: Additional file 1 — Table 1: Patient selection based on the antipsychotic prescription refilling records. The patient selection process for data analysis. [file 1471-244X-9-80-S1.DOC]

Table 1: Patient selection based on the antipsychotic prescription refilling records

|  |  | # of patients |
| --- | --- | --- |
| VISN 16 patients | Diagnosis of schizophrenia* (10/ 2002-09/2005) | 8,816 |
| Exclude criterion # 1 | Patent received ≥2 index drugs on the same day | 677 |
| Exclude criterion # 2 | Patients took an antipsychotic agent once only | 393 |
| Exclude criterion # 3 | Patients without a new treatment episode | 2,354 |
| Exclude criterion # 4 | Patients changed to FGA | 683 |
| Study Population |  | 4,709 |
| Mon(+) | Received metabolic monitoring | 3,568 (75.77%) |
| Mon(-) | Did not receive metabolic monitoring | 1,141 (24.23%) |

Schizophrenia: Primary diagnosis in 1 inpatient service or at least 2 outpatient visits based on the International Classification of Diseases, 9th Revision, Clinical Modification (ICD-9-CM): 295.xx
