# Supplementary material for: Predictors of metabolic monitoring among schizophrenia patients with a new episode of second-generation antipsychotic use in the Veterans Health Administration
Source: BMC Psychiatry. 2009 Dec 18;9:80. doi: 10.1186/1471-244X-9-80 (PMC2807859; doi:10.1186/1471-244X-9-80)
Supplement: Additional file 2 — Table 2: Baseline characteristics and health services utilization for schizophrenia patients with versus without baseline monitoring. Cross-cohort comparisons of baseline characteristics and health services utilization. [file 1471-244X-9-80-S2.DOC]

**Table 2: Baseline characteristics and health services utilization for schizophrenia patients with versus without baseline monitoring**

|  | Mon(+) | | | | | | | | | | | | | | | | | | | | | | | | | | | | | | Mon(-) | | | | | | | | | | | | | | | | | | | | | | | | P value | | |
| --- | --- | --- | --- | --- | --- | --- | --- | --- | --- | --- | --- | --- | --- | --- | --- | --- | --- | --- | --- | --- | --- | --- | --- | --- | --- | --- | --- | --- | --- | --- | --- | --- | --- | --- | --- | --- | --- | --- | --- | --- | --- | --- | --- | --- | --- | --- | --- | --- | --- | --- | --- | --- | --- | --- | --- | --- | --- |
|  | New start | | | | | | | Switch | | | | | | | | Augmentation | | | | | | | All Episodes | | | | | | | | New start | | | | | Switch | | | | | | | Augmentation | | | | | | All Episodes | | | | | |
|  | N | | | % | | | | N | | | | | | % | | N | | | | % | | | N | | | % | | | | | N | | | % | | | | N | | | % | | | N | | | % | | | N | | % | | |  | | |
| Sample size | 2,815 | | | 78.90 | | | | 439 | | | | | | 12.30 | | 314 | | | | 8.80 | | | 3,568 | | | 75.77 | | | | | 986 | | | 86.42 | | | | 97 | | | 8.50 | | | 58 | | | 5.08 | | | 1,141 | | 24.23 | | |  | | |
| Age( SD) | 55.66 (10.70) | | | | | | | 53.74 (10.78) | | | | | | | | 54.05 (9.40) | | | | | | | 55.28 (10.62) | | | | | | | | 54.54 (12.53) | | | | | | | 53.19 (12.93) | | | | | | 54.76 (12.64) | | | | | | 54.44 (12.56) | | | | | 0.0407 | | |
| Male | 2,645 | | | 93.96 | | | | 401 | | | | | | 91.34 | | 296 | | | | 94.27 | | | 3,342 | | | 93.67 | | | | | 919 | | | 93.20 | | | | 85 | | | 87.63 | | | 55 | | | 94.83 | | | 1,059 | | 92.81 | | | 0.3106 | | |
| Race | | | | | | | | | | | | | | | | | | | | | | | | | | | | | | | | | | | | | | | | | | | | | | | | | | | | | | | 0.1796 | | |
| Black | 1,368 | | | 48.60 | | | | 172 | | | | | 39.18 | | | | 140 | | | 44.59 | | | 1,680 | | | | 47.09 | | | | 514 | | | 52.13 | | | | 43 | | | 44.33 | | | | 25 | | | 43.10 | | | 582 | | 51.01 | | |  | |
| White | 1,268 | | | 45.04 | | | | 249 | | | | | 56.72 | | | | 158 | | | 50.32 | | | 1,675 | | | | 46.95 | | | | 412 | | | 41.78 | | | | 50 | | | 51.55 | | | | 31 | | | 53.45 | | | 493 | | 43.21 | | |  | |
| Others | 179 | | | 6.36 | | | | 18 | | | | | 4.29 | | | | 16 | | | 5.1 | | | 213 | | | | 5.96 | | | | 60 | | | 6.09 | | | | 4 | | | 4.12 | | | | 2 | | | 3.44 | | | 66 | | 5.78 | | |  | |
| Non-VA Insurance | 1,082 | | | | 38.44 | | | | 197 | | | | | 44.87 | | | | 134 | | | 42.68 | | | 1,413 | | | | 39.60 | | | | 323 | | | 32.76 | | | | 27 | | | 27.84 | | | 19 | | | 32.76 | | | 369 | | 32.34 | | | <0.0001 | |
| CCI Score | 1.22 (0.68) | | | | | | | | 1.21 (0.60) | | | | | | | | 1.12 (0.58) | | | | | | 0.67 (1.21) | | | | | | | | 0.45 (0.93) | | | | | | | 0.56 (1.38) | | | | | | | 7.00 (0.73) | | | | | | 0.46 (0.96) | | | | | <0.0001 | |
| Diagnosis of substance dependence disorders | | | | | | | | | | | | | | | | | | | | | | | | | | | | | | | | | | | | | | | | | | | | | | | | | | | | | | | <0.0001 | | |
| Yes | 1,275 | | | 45.29 | | | | | | | 209 | | | 47.61 | | | 133 | | | 42.36 | | 1,617 | | | | 45.32 | | | | | 357 | | | 36.21 | | | | 33 | | | 34.02 | | | | 18 | | | 31.03 | | | 408 | | 35.76 | | |  | |
| Office visits |  | | |  | | | | | | |  | | |  | | |  | | |  | |  | | | |  | | | | |  | | |  | | | |  | | |  | | | |  | | |  | | |  | |  | | |  | |
| Psychiatric(N) | 8.31(19.2) | | | | | | | | | | 10.82(19.89) | | | | | | 13.62(26.18) | | | | | 9.08 (20.06) | | | | | | | | | 5.56(11.58) | | | | | | | 7.74(12.24) | | | | | | | 7(10.04) | | | | | | 5.82(11.58) | | | | | <0.0001 | |
| Psychiatric(Y) | 14.76(19.95) | | | | | | | | | | 13.78(17.11) | | | | | | 12.62(16.78) | | | | | 14.45(19.37) | | | | | | | | | 10.18(18.53) | | | | | | | 9.53(13.95) | | | | | | | 8.4(10.31) | | | | | | 10.03(17.85) | | | | | <0.0001 | |
| Total | 23.06(27.98) | | | | | | | | | | 24.6(25.57) | | | | | | 26.24(32.09) | | | | | 23.53(28.1) | | | | | | | | | 15.74(22.14) | | | | | | | 17.27(18.29) | | | | | | | 15.4(16) | | | | | | 15.85(21.56) | | | | | <0.0001 | |
| Number of hospital admissions during 180 days prior to baseline monitoring services | | | | | | | | | | | | | | | | | | | | | | | | | | | | | | |  | | |  | | | |  | | |  | | |  | | |  | | |  | |  | | |  | | |
|  | 0.68 (1.25) | | | | | | | | | | 1 (1.52) | | | | | | 0.46 (0.99) | | | | | 0.7 (1.27) | | | | | | | | | 0.35 (0.81) | | | | | | | 0.71 (1.15) | | | | | | | 0.41 (1.04) | | | | | | 0.38 (0.86) | | | | | <0.0001 | |
| Mean length (days) of hospital for all admissions during the pre-existing year | | | | | | | | | | | | | | | | | | | | | | | | | | | | | | |  | | | | | | |  | | | | | |  | | | | | |  | | | | |  | | |
| mean (SD) | 12.99 (32.14) | | | | | | | | | | 17.04(31.67) | | | | | | 6.96 (16.58) | | | | | 12.96 (31.11) | | | | | | | | | 6.21 (18.79) | | | | | | 8.48 (14.72) | | | | | | | | 5.67 (12.26) | | | | | | 6.37(18.20) | | | | | <0.0001 | |
| Diagnosis based on ICD-9-CM codes during the pre-existing year | | | | | | | | | | | | | | | | | | | | | | | | | | | | | | |  | | | | | |  | | | | | | |  | | | | | |  | | | | |  | | |
| Diabetes | | 715 | | | 25.40 | | | | | | 118 | 26.88 | | | | | 80 | | 25.48 | | | | 913 | | | | | | 25.59 | | 79 | | 8.01 | | | | 11 | | | 11.34 | | | | | | 5 | | 8.62 | | 95 | | | | 8.33 | | | <0.0001 |
| Dyslipidemia | | 988 | | | 35.10 | | | | | | 147 | 33.49 | | | | | 125 | | 39.81 | | | | 1,260 | | | | | | 35.31 | | 125 | | 12.68 | | | | 10 | | | 10.31 | | | | | | 2 | | 3.45 | | 137 | | | | 12.01 | | | <0.0001 |
| Hypertension | | 1,461 | | | 51.90 | | | | | | 213 | 48.52 | | | | | 154 | | 49.04 | | | | 1,828 | | | | | | 51.23 | | 309 | | 31.34 | | | | 28 | | | 28.87 | | | | | | 18 | | 31.03 | | 355 | | | | 31.11 | | | <0.0001 |
| Medication use during the pre-existing year | | | | | | | | | | | | | | | | | | | | | | | | | | | | | | |  | |  | | | |  | | |  | | | | | |  | |  | |  | | | |  | | |  |
| Anti-DM | | 512 | | | | 18.19 | | | 88 | | | | | 20.05 | | | | 63 | | 13.27 | | | | | 663 | | | | | 18.58 | 43 | | | 4.36 | | | 11 | | | 11.34 | | | | | | 2 | | 3.45 | | 56 | | | | 4.91 | | | <0.0001 |
| Anti-HTN | | 1,562 | | | | 55.49 | | | 236 | | | | | 53.76 | | | | 166 | | 52.87 | | | | | 1,964 | | | | | 55.04 | 344 | | | 34.89 | | | 33 | | | 34.02 | | | | | | 23 | | 39.66 | | 400 | | | | 35.06 | | | <0.0001 |
| Anti-Lipid | | 806 | | | | 28.63 | | | 119 | | | | | 27.11 | | | | 98 | | 31.21 | | | | | 1,023 | | | | | 28.67 | 107 | | | 10.85 | | | 7 | | | 7.22 | | | | | | 5 | | 8.62 | | 119 | | | | 10.43 | | | <0.0001 |
| Year 2003 | | | 1,002 | | | | 35.60 | | | 168 | | | | | 38.27 | | | 137 | | 43.63 | | | | | 1,307 | | | | | 36.63 | | 391 | | 39.66 | | | 42 | | | 43.30 | | | | | | 34 | | 58.62 | | 467 | | | | 40.93 | | | <0.0001 |
| Year 2004 | | | 1,157 | | | | 41.10 | | | 151 | | | | | 34.40 | | | 107 | | 34.08 | | | | | 1,415 | | | | | 39.66 | | 371 | | 37.63 | | | 27 | | | 27.84 | | | | | | 16 | | 27.59 | | 414 | | | | 36.28 | | |  |
| Year 2005 | | | 626 | | | | 22.24 | | | 101 | | | | | 23.01 | | | 51 | | 16.24 | | | | | 778 | | | | | 21.80 | | 192 | | 19.47 | | | 18 | | | 18.56 | | | | | | 2 | | 3.45 | | 212 | | | | 18.58 | | |  |

Augmentation: concurrent use of an SGA and previous antipsychotic(s) for longer than 60 days

New start: Receiving an index SGA agent without any antipsychotics in prior 60 days

Switch: discontinuation of the previous antipsychotic agent within 60 days after the index date

SD: standard deviation; P value between Mon+ and Mon- groups; CCI: Charlson Comorbidity Index; Anti-DM: Anti-diabetics; Anti-HTN: Anti-hypertensive; Anti-Lipid: Lipid lowering drugs
