# Supplementary material for: Predictors of metabolic monitoring among schizophrenia patients with a new episode of second-generation antipsychotic use in the Veterans Health Administration
Source: BMC Psychiatry. 2009 Dec 18;9:80. doi: 10.1186/1471-244X-9-80 (PMC2807859; doi:10.1186/1471-244X-9-80)
Supplement: Additional file 3 — Table 3: Baseline antipsychotic use patterns for schizophrenia patients with versus without baseline antipsychotic metabolic monitoring. Cross-cohort comparisons of use patterns of baseline antipsychotic agents. [file 1471-244X-9-80-S3.DOC]

**Table 3: Baseline antipsychotic use patterns for schizophrenia patients with versus without baseline antipsychotic metabolic monitoring**

|  | Mon(+) | | | | | | Mon(-) | | | | P value |
| --- | --- | --- | --- | --- | --- | --- | --- | --- | --- | --- | --- |
|  | New start (N=2,784) | | Switch  (N=428) | Augmentation (N=293) | | All Episodes | New start (N=973) | Switch  (N=96) | Augmentation (N=54) | All Episodes |
|  | Mean (SD) | | Mean (SD) | Mean (SD) | Mean (SD) | | Mean (SD) | Mean (SD) | Mean (SD) | Mean (SD) |
| Duration of AP use^1^ | | 232.69 (237.64) | 144.22 (216.15) | 83.13  (147.50) | 208.65  (233.56) | | 163.31  (205.77) | 146.06 (235.32) | 127.69 (189.29) | 160.03  (207.65) | <0.0001 |
| MPR^2^ | 0.52 (0.38) | | 0.74 (0.50) | 0.98 (0.67) | 0.59 (0.45) | | 0.44 (0.34) | 0.61 (0.51) | 0.72 (0.44) | 0.47 (0.37) | <0.0001 |
| FGAs^3^ | 0.10 (0.31) | | 0.26 (0.45) | 0.35 (0.48) | 0.14 (0.35) | | 0.08 (0.27) | 0.28 (0.45) | 0.38 (0.49) | 0.11 (0.31) | 0.0032 |
| SGAs^4^ | 1.38 (0.54) | | 1.93 (0.56) | 1.68 (0.51) | 1.48 (0.57) | | 1.29 (0.45) | 1.78 (0.44) | 1.62 (0.49) | 1.35 (0.48) | <0.0001 |
| FGA+SGA^5^ | 1.48 (0.59) | | 2.20 (0.48) | 2.03 (0.24) | 1.62 (0.61) | | 1.36 (0.49) | 2.06 (0.24) | 0 (0) | 1.46 (0.51) | <0.0001 |

Augmentation: concurrent use of an SGA and previous antipsychotic(s) for longer than 60 days

New start: receiving an index SGA without any antipsychotics in prior 60 days

Switch: discontinuation of the previous antipsychotic agent within 60 days after the index date

1: Duration of use of the most recent antipsychotic during the prior year;

2: MPR: Medication possession ratio in the prior year;

3: Number of FGA during the prior year;

4: Number of different SGA during the prior year;

5: Number of different SGA and FGA antipsychotics during the prior year.

P value between Mon+ and Mon- groups
