# Supplementary material for: Predictors of metabolic monitoring among schizophrenia patients with a new episode of second-generation antipsychotic use in the Veterans Health Administration
Source: BMC Psychiatry. 2009 Dec 18;9:80. doi: 10.1186/1471-244X-9-80 (PMC2807859; doi:10.1186/1471-244X-9-80)
Supplement: Additional file 4 — Table 4: Baseline metabolic syndrome parameters among patients with schizophrenia receiving a new treatment episode of SGA. Cross-cohort comparisons of baseline metabolic syndrome parameters. [file 1471-244X-9-80-S4.DOC]

**Table 4: Baseline metabolic syndrome parameters among patients with schizophrenia receiving a new treatment episode of SGA**

|  | Mon(+) | | | | | | | | Mon(-) | | | | | | | |
| --- | --- | --- | --- | --- | --- | --- | --- | --- | --- | --- | --- | --- | --- | --- | --- | --- |
|  | New start | | Switch | | Augmentation | | All Episodes | | New start | | Switch | | Augmentation | | All Episodes | |
|  | N | % | N | % | N | % | N | % | N | % | N | % | N | % | N | % |
| Total | 2,815 | 78.90 | 439 | 12.30 | 314 | 8.80 | 3,568 | 75.77 | 986 | 86.42 | 97 | 8.50 | 58 | 5.08 | 1,141 | 24.23 |
| **HDL** |  |  |  |  |  |  |  |  |  |  |  |  |  |  |  |  |
| <40 or 50 (M, F) | 1,048 | 37.23 | 165 | 37.59 | 115 | 36.62 | 1,328 | 37.22 | - | - | - | - | - | - | - | - |
| ≥40 or 50 (M, F) | 1,180 | 41.92 | 182 | 41.46 | 128 | 40.76 | 1,490 | 41.76 | - | - | - | - | - | - | - | - |
| Missing | 587 | 20.85 | 92 | 20.96 | 71 | 22.61 | 750 | 21.02 | - | - | - | - | - | - | - | - |
| **Glucose** |  |  |  |  |  |  |  |  |  |  |  |  |  |  |  |  |
| ≥ 110 mg/dl | 330 | 11.72 | 57 | 12.98 | 43 | 13.69 | 430 | 12.05 | - | - | - | - | - | - | - | - |
| < 110 mg/dl | 742 | 26.36 | 125 | 28.47 | 86 | 27.39 | 953 | 26.71 | - | - | - | - | - | - | - | - |
| Missing | 1,743 | 61.92 | 257 | 58.54 | 185 | 58.92 | 2,185 | 61.24 |  |  |  |  |  |  |  |  |
| **Triglyceride** |  |  |  |  |  |  |  |  |  |  |  |  |  |  |  |  |
| ≥ 150 mg/dl | 345 | 12.26 | 53 | 12.07 | 30 | 9.55 | 428 | 12.00 | - | - | - | - | - | - | - | - |
| < 150 mg/dl | 552 | 19.61 | 69 | 15.72 | 43 | 13.69 | 664 | 18.61 | - | - | - | - | - | - | - | - |
| Missing | 1,918 | 68.13 | 317 | 72.21 | 241 | 76.75 | 2,476 | 69.39 |  |  |  |  |  |  |  |  |
| **Blood Pressure (p=.5486)** | | | |  |  |  |  |  |  |  |  |  |  |  |  |  |
| ≥ 130/85 mm/Hg | 1,475 | 52.40 | 202 | 46.01 | 180 | 57.32 | 1,857 | 52.05 | 369 | 37.42 | 47 | 48.45 | 31 | 53.45 | 447 | 39.18 |
| < 130/85 mm/Hg | 1,264 | 44.90 | 225 | 51.25 | 126 | 40.13 | 1,615 | 45.26 | 354 | 35.90 | 39 | 40.21 | 14 | 24.14 | 407 | 35.67 |
| Missing | 76 | 2.70 | 12 | 2.73 | 8 | 2.55 | 96 | 2.69 | 263 | 26.67 | 11 | 11.34 | 13 | 22.41 | 287 | 25.15 |
| Missing |  |  |  |  |  |  |  |  |  |  |  |  |  |  |  |  |
| **BMI (p<.0001)^1^** | | | |  |  |  |  |  |  |  |  |  |  |  |  |  |
| <18.5 | 44 | 1.56 | 7 | 1.59 | 2 | 0.64 | 53 | 1.49 | 15 | 1.52 | 0 | 0.00 | 0 | 0.00 | 15 | 1.31 |
| 18.5 – 24.9 | 579 | 20.57 | 79 | 18.00 | 52 | 16.56 | 710 | 19.90 | 164 | 16.63 | 16 | 16.49 | 8 | 13.79 | 188 | 16.48 |
| 25.0 – 29.9 | 771 | 27.39 | 106 | 24.15 | 71 | 22.61 | 948 | 26.57 | 190 | 19.27 | 26 | 26.80 | 9 | 15.52 | 225 | 19.72 |
| ≥30.0 | 895 | 31.79 | 172 | 39.18 | 129 | 41.08 | 1,196 | 33.52 | 134 | 13.59 | 20 | 20.62 | 10 | 17.24 | 164 | 14.37 |
| Missing | 526 | 18.69 | 75 | 17.08 | 60 | 19.11 | 661 | 18.53 | 483 | 48.99 | 35 | 36.08 | 31 | 53.45 | 549 | 48.12 |
| **BMI (p<.0001)^2^** |  |  |  |  |  |  |  |  |  |  |  |  |  |  |  |  |
| <28.8 | 1,224 | 43.48 | 169 | 38.50 | 107 | 34.08 | 1,500 | 42.04 | 327 | 33.16 | 38 | 39.18 | 15 | 25.86 | 380 | 33.30 |
| ≥28.8 | 1,065 | 37.83 | 195 | 44.42 | 147 | 46.82 | 1,407 | 39.43 | 176 | 17.85 | 24 | 24.74 | 12 | 20.69 | 212 | 18.58 |
| Missing | 526 | 18.69 | 75 | 17.08 | 60 | 19.11 | 661 | 18.53 | 485 | 48.99 | 35 | 36.08 | 31 | 53.45 | 549 | 48.12 |
| **Number of Positive Metabolic Syndrome Parameters (Method 1)^3^** | | | | | | | | | | |  |  |  |  |  |  |
| >= 3 | 478 | 22.03 | 72 | 21.30 | 73 | 30.67 | 623 | 22.69 | 0 | 0 | 0 | 0 | 0 | 0 | 0 | 0 |
| < 3 | 1,692 | 77.97 | 266 | 78.70 | 165 | 69.33 | 2,123 | 77.31 | 986 | 100 | 97 | 100 | 58 | 100 | 1,141 | 100 |
| **Number of Positive Metabolic Syndrome Parameters (Method 2)^4^** | | | | | | | | | | |  |  |  |  |  |  |
| >= 3 | 1,072 | 38.08 | 176 | 40.09 | 139 | 44.27 | 1,387 | 38.87 | 68 | 6.90 | 10 | 10.31 | 4 | 6.90 | 82 | 7.19 |
| < 3 | 1.743 | 61.92 | 263 | 59.91 | 175 | 55.73 | 2,181 | 61.13 | 918 | 93.10 | 87 | 89.69 | 54 | 93.10 | 1,059 | 92.81 |

Augmentation: concurrent use of an SGA and previous antipsychotic(s) for longer than 60 days

New start: receiving an index SGA without any antipsychotics in prior 60 days

Switch: discontinuation of the previous antipsychotic agent within 60 days after the index date

1. http://www.nhlbisupport.com/bmi: <18.5: underweight; 18.5–24.9: normal weight; 25.0-29.9: overweight; ≥30.0: obese
2. BMI ≥28.8 is equivalent to waist circumference of more than 102 cm (>40 inches) for men and >88cm (>35 inches) for women.
3. Method 1: Samples were required to have all 5 lab-testing-based indicators without missing values.
4. Method 2: Samples were required to have all 5 composite indicators measured by lab testing, diagnosis codes, and medication use.
