# Supplementary material for: Predictors of metabolic monitoring among schizophrenia patients with a new episode of second-generation antipsychotic use in the Veterans Health Administration
Source: BMC Psychiatry. 2009 Dec 18;9:80. doi: 10.1186/1471-244X-9-80 (PMC2807859; doi:10.1186/1471-244X-9-80)
Supplement: Additional file 5 — Table 5: Logistic regression stepwise model to identify predictors of undergoing baseline antipsychotic metabolic monitoring. Influencing factors identified using a logistic regression on whether a patient receiving baseline antipsychotic metabolic monitoring. [file 1471-244X-9-80-S5.DOC]

**Table 5: Logistic regression stepwise model to identify predictors of undergoing baseline antipsychotic metabolic monitoring**

| Variable | Estimate | Standard Error | Odds Ratio | 95% Confidence Interval | | P value | |
| --- | --- | --- | --- | --- | --- | --- | --- |
| Intercept | -0.7852 | 0.1275 |  |  |  | | <0.0001 |
| Switch (reference: new start) | 0.1600 | 0.1375 | 1.173 | 0.896 | 1.537 | | 0.2447 |
| Augmentation (reference: new start) | 0.4571 | 0.1642 | 1.580 | 1.145 | 2.179 | | 0.0054 |
| # of non-psychiatric outpatient visits | 0.00739 | 0.00299 | 1.007 | 1.002 | 1.013 | | 0.0136 |
| Total length of stay (days) in the pre-existing year | 0.0111 | 0.00211 | 1.011 | 1.007 | 1.015 | | <0.0001 |
| # of different FGA^1^ | 0.3603 | 0.1219 | 1.434 | 1.129 | 1.821 | | 0.0031 |
| # of different SGA^2^ | 0.4074 | 0.0778 | 1.503 | 1.290 | 1.751 | | <0.0001 |
| Medication duration (days) of most recent antipsychotic | 0.000902 | 0.000181 | 1.001 | 1.001 | .1.001 | | <0.0001 |
| Diagnosis of substance dependence disorder | 0.3785 | 0.0763 | 1.460 | 1.257 | 1.696 | | <0.0001 |
| Baseline BMI^3^ | 0.7188 | 0.0890 | 2.052 | 1.724 | 2.443 | | <0.0001 |
| Baseline diabetes diagnosis or medication | 0.8482 | 0.1201 | 2.336 | 1.846 | 2.955 | | <0.0001 |
| Baseline dyslipidemia diagnosis or medication | 0.8916 | 0.0940 | 2.439 | 2.029 | 2.932 | | <0.0001 |
| Baseline hypertension diagnosis or medication | 0.4037 | 0.0773 | 1.497 | 1.287 | 1.743 | | <0.0001 |

Augmentation: concurrent use of an SGA and previous one for longer than 60 days

New start: receiving an index SGA without antipsychotics in prior 60 days

Switch: discontinuation of the previous antipsychotic agent within 60 days after the index date

1: Number of different FGA during the pre-existing year

2: Number of different SGA in the pre-existing year

3: Baseline BMI=1 if BMI ≥28.8, which is equivalent to waist circumference of more than 102 cm (>40 inches) for men and >88cm (>35 inches) for women.
